# Glucagon-like peptide-1 receptor agonists (GLP-1RAs) for the treatment of type 2 diabetes mellitus: friends or foes to bone health? a narrative review of clinical studies

**DOI:** 10.1007/s12020-025-04253-4

**Published:** 2025-05-08

**Authors:** Antonella Al Refaie, Leonardo Baldassini, Caterina Mondillo, Sara Gonnelli, Elena Ceccarelli, Roberto Tarquini, Stefano Gonnelli, Luigi Gennari, Carla Caffarelli

**Affiliations:** 1 Section of Internal Medicine, Department of Medicine, Surgery and Neuroscience, University of Siena, Siena, Italy; 2 Division of Internal Medicine I, San Giuseppe Hospital, Empoli, Italy; 3 Department of Geriatrics, University Hospital of Nice, Nice, France

**Keywords:** Glucagon-like peptide-1 receptor agonists (GLP-1RAs), Type 2 diabetes mellitus (T2DM), Bone mineral density (BMD), Trabecular bone score (TBS), Bone turnover markers (BTMs), Fragility fractures

## Abstract

Glucagon-like peptide-1 receptor agonists (GLP-1RAs) are a relatively new class of drugs for treatment of Type 2 Diabetes mellitus (T2DM). They have proven to be excellent drugs not only for the results on glycemic control but also for weight loss, cardiovascular protection and several other potential metabolic effects. In contrast, the effects of GLP-1RAs drugs on bone metabolism and bone mineral density (BMD) remain less clearly defined. This narrative review aimed to explore the relationship between GLP-1RAs and bone in T2DM patients by reviewing clinical studies which assessed the effects of GLP-1RAs on BMD, markers of bone turnover and fragility fractures. In vitro and animal studies have demonstrated that GLP-1RAs treatment promotes bone formation and inhibits bone resorption. However, in humans, GLP-1RAs therapy has been shown to primarily stimulate bone resorption, as evidenced by a significant increase in type I collagen C-terminal cross-linked telopeptide levels, while promoting new bone formation to a lesser extent. Clinical studies indicate that GLP-1RAs therapy, in both diabetic and non-diabetic patients, results in a reduction in BMD, which is more pronounced at skeletal sites subjected to higher mechanical loading, such as the femur and tibia, and appears to correlate with the degree of weight loss. Furthermore, in the studies reviewed, parameters related to bone quality and strength, such as Trabecular bone score (TBS), microindentation, High-resolution peripheral Quantitative Computed Tomography (HR-pQCT), and Radiofrequency Echographic Multi Spectrometry (REMS) remain unaffected by GLP-1RAs. Additionally, the incidence of fragility fractures does not increase.

## Introduction

Type 2 Diabetes mellitus (T2DM), the most common chronic metabolic disorder worldwide, negatively impacts bone health, making diabetic patients more susceptible to fractures than the general population [1]. Moreover, the heightened incidence of fractures diminishes quality of life and increases mortality risk in individuals with diabetes [2]. Consequently, preventing diabetic osteopathy is of paramount importance. Growing awareness of diabetic osteopathy’s importance has prompted studies on the effects of antidiabetic drugs on bone health. Currently, metformin appears to have a neutral effect on bone, thiazolidinediones are known to reduce bone density, insulin slightly increases fracture risk (likely due to a higher incidence of falls), while sodium-glucose cotransporter 2 (SGLT-2) inhibitors appear to have a neutral effect on bone, but studies have not yet reached a consensus [3]. Glucagon-like peptide-1 receptor agonists (GLP-1RAs) are a relatively new class of drugs revolutionizing the treatment of T2DM, so that in the latest guidelines, GLP-1RAs are even recommended as a first-line treatment for T2DM patients with cardiovascular disease, renal insufficiency, or overweight/obesity [4, 5]. Beyond their effectiveness in controlling blood glucose levels, GLP-1RAs offer a range of additional benefits, including weight loss and reduced risk of cardiovascular events [4, 5].

Recently, there has been growing interest in the effects of GLP-1RAs on bone health. Several studies, primarily conducted in vitro and in animal models, suggest that GLP-1RAs may positively impact bone metabolism by promoting osteoblast differentiation and proliferation [6]. GLP-1RAs directly stimulate bone metabolism, as well as β-catenin, GSK-3β, and T cell factor activity. By modulating β-catenin signal transduction, GLP-1RAs encourage the osteogenic differentiation of bone marrow stromal cells [7]. Moreover, in rat models, the upregulation of RUNX2, alkaline phosphatase (ALP), collagen type I (COL1), osteocalcin (OC), and the N-terminal propeptide of procollagen type I (P1NP)-all of which are involved in osteoblast stimulation—appears to be facilitated by GLP-1RAs [8]. Furthermore, GLP-1RAs reduce bone resorption both by increasing the expression of osteoprotegerin (OPG) gene and by stimulating thyroid C cells to release calcitonin, a hormone that inhibits osteoclastic bone resorption [9]. Therefore, GLP-1RAs can inhibit osteoclast activity resulting in a reduction in serum levels of the type I collagen C-terminal cross-linked telopeptide (CTX) and the urine deoxypyridinoline (DPD)/creatinine ratio [9]. Furthermore, studies conducted on ovariectomized mice and rats have reported that administering GLP-1RAs increases bone mineral density (BMD) and appears to have a protective effect on bone microstructure, particularly enhancing trabecular thickness and area [10, 11].

While in vitro and animal studies suggest that GLP-1 receptor agonists (GLP-1RAs) may enhance bone health by reducing bone resorption, stimulating new bone formation, and increasing bone mineral density (BMD), human studies, particularly in individuals with T2DM, have yielded conflicting results regarding bone markers, BMD, and fragility fractures [12–14].

This narrative review aims to explore the relationship between GLP-1RAs and bone health in T2DM patients by reviewing clinical studies which assessed the effects of GLP-1RAs therapies on BMD, markers of bone turnover and fragility fractures.

## Materials and methods

**Fig. 1:**
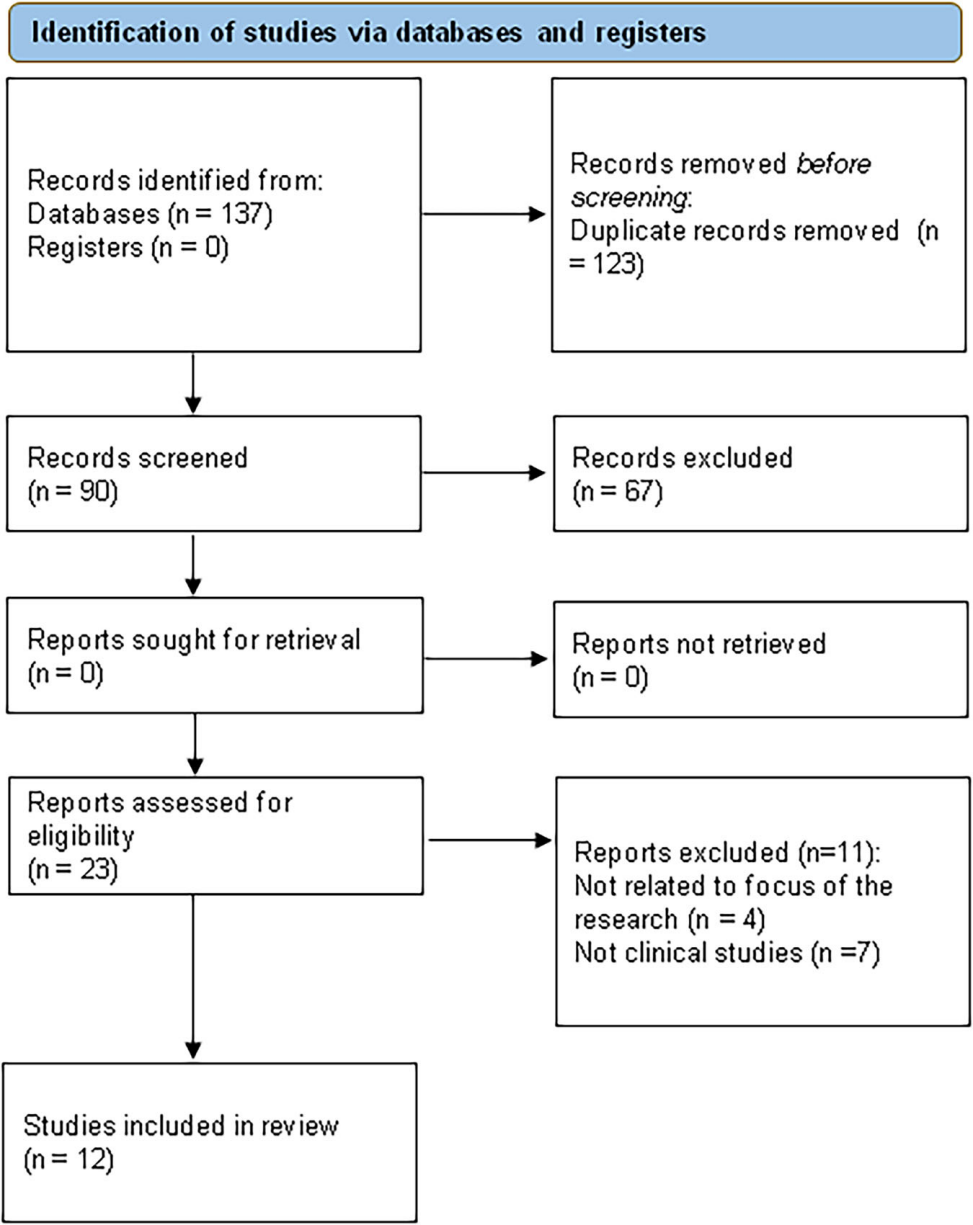
Flow chart of the studies identified and included in the narrative review

A review of the literature was done from the inception to December 2024. The following terms were used to search the databases of Pubmed-Medline, Cochrane Library, ClinicalTrials.gov, and SCOPUS: “GLP-1RAs” or “GLP1 receptor agonists” AND “bone mineral density” or “bone metabolism” AND “clinical studies.” Using these search terms across the above mentioned databases 137 results were found. The titles, abstracts and complete texts were screened separately. We conducted a screening process, primarily looking at publications which aligned with clinical research on GLP-1RAs effects on bone. Duplicates, meta-analyses and reviews were then eliminated. At the end only 12 records were included in qualitative and quantitative synthesis. The process of selecting the studies for review in adherence with the PRISMA 2020 process is shown in Fig. 1.

## Results

The studies selected to evaluate the effects of GLP-1RAs therapies on bone status are summarized in Table 1 [15–26]. Except for two studies which analyze national registries, the remaining studies focus on patient populations ranging from 15 to 132 individuals. In particular 10 studies focus on patients with type 2 diabetes, 1 study examines those with type 1 diabetes, and 1 study by Hansen involves patients with T2DM and osteopenia. In the 12 studies selected for this review, the primary focus was on evaluating the effects of GLP-1RAs therapy on BMD, bone turnover markers, and, when possible, fragility fractures.

**Table 1:** Main characteristics of the studies

Study/years/country	Subjects	Study /duration	Assessment	Results
Bunck MC et al. Finland [15]	T2DM Met + Exenatide = 36 Met + Ins Glargine = 33	Randomised Clinical Study (44 weeks)	b-ALP BMD-LS and BMD-FN and BMD-TH by DXA	Body Weight: Met + exenatide = −3.5Kg ** Met + ins glargine = +0.3 Kg b-ALP: n.s.c. BMD: n.s.c.
Li R. et al. China [16]	T2DM Exenatide = 20 Insul lispro = 21 Piglitazone = 21	Randomised parallel group clinical trial (24 weeks)	CTX TRAcP5b BMD-LS BMD-Hip	Body Weight: Exenatide = −4.7Kg ** Insul lispro = −0.7Kg Piglitazone = −1.0Kg CTX: n.s.c. TRAcP5b: n.s.c. BMD-LS and BMD-hip: n.s.c.
Driessen JH et al (2015) Netherland [17]	T2DM GLP-1RAs (Exenatide or Liraglutide) = 8.354	Population-based cohort study (5.1 years)	Fractures	Fractures = 122 HR (95% CI) 0.97 (0.71–1.31) Exenatide = 65 fractures Liraglutide = 57 fractures
Gilbert MP et al. USA [18]	T2DM Liraglutide 1.2 mg = 20 Liraglutide 1.8 mg = 23 Glimepiride = 18	Subgroup of participants to LEAD-3, a double-blind, active control, phase III, multicenter trial, (52 weeks)	b-ALP WB-BMD	b-ALP: n.s.c. WB-BMD: n.s.c.
Hygum K et al. Denmark [19]	T2DM Liraglutide 1.8 mg = 30 Placebo = 30	Randomized, double-blinded, placebo-controlled, prospective, clinical trial (26 weeks)	CTX P1NP b-ALP BMD-LS and BMD-FN and BMD-TH by DXA HRpQCT tibia and radius QCT spine and hip	Body Weight: Liraglutide: −3.8Kg ** at 13 weeks Placebo: 0.06 Kg CTX: Liraglutide:+0.07*; Placebo : +0.06* P1NP: n.s.c. b-ALP: Liraglutide:+0.07; Placebo : +0.06 BMD-LS, BMD-FN, BMD-TH: n.s.c. HRpQCT: n.s.c. QCT spine and hip: n.s.c.
Cai TT et al. China [20]	T2DM Exenatide = 19 Dulaglutide = 19 Glargine = 10 Placebo = 17	Single-blinded study (52weeks)	BMD-LS and BMD-FN and BMD-TH by DXA	Body Weight: Exenatide: −1.5 Kg Dulaglutide: +0.08 Kg Glargine: +2 kg Placebo: +1 kg BMD-LS: glargine: + 0.030 (g/cm^2^)* BMD-FN: dulaglutide: −0.010 (g/cm^2^)* BMD-TH: exenatide : +0.080(g/cm^2^)
Johansen NJ et al. (2021) Denmark [21]	T1DM Exenatide = 53 Placebo= 52	Randomized, double-blinded, parallel-group trial (26 weeks)	CTX P1NP BMD-LS and BMD-FN and BMD-TH by DXA	Body Weight: Exenatide: −4.4 Kg ** CTX and P1NP: n.s.c. BMD-LS: n.s.c. BMD-FN: n.s.c. BMD-TH: n.s.c.
Al-Mashhadi ZK et al. (2022) Denmark [22]	T2DM GLP-1RAs+met = 16723 DPP4 + met = 26093	Population-based cohort study using Danish national health registries (600 days)	MOF (hip, vertebrae, homerus, forearm)	Hip fracture: HR 0.65 [0.46 – 0.93] MOF:HR 0.86 [0.73–1.03]
Akyay OZ et al. Turkey [23]	T2DM Exenatide = 15 Glargine = 15	Randomized, controlled, open-label, 2-arm parallel-group study (24 weeks)	b-ALP NTX BMD-LS and BMD-FN and BMD-TH by DXA	b-ALP: n.s.c. NTX: n.s.c. BMD-LS: n.s.c. BMD-FN: n.s.c. BMD-TH: n.s.c.
Huang CF et al. China [24]	T2DM + OP DPP-4i to GLP-1RAs = 132 DPP-4i to DPP-4i = 133	Retrospective cohort study (3-4 years)	BMD-LS and BMD-FN and BMD-TH by DXA	Body Weight: DPP-4i to GLP-1RA: −2.25 Kg * DPP-4i to DPP-4i: −0.81 Kg DPP-4i to GLP-1RA:BMD-LS: −0.028 (g/cm^2^)* BMD-Hip: n.s.c. DPP-4i to DPP-4i: BMD-LS: n.s.c. BMD-Hip: n.s.c.
Hansen MS et al. Denmark [25]	T2DM Semaglutide = 32 Placebo = 32	Randomised, placebo-controlled, double-blinded, phase 2 clinical trial (52 weeks)	P1NP CTX BMD-LS and BMD-FN and BMD-TH by DXA HRpQCT tibia and ra-dius	Body Weight: −6.8Kg ** P1NP: n.s.c. CTX: +166 ng/L** BMD-LS: −0,018 (g/cm^2^)* BMD-TH: −0,020 (g/cm^2^)* BMD-FN: n.s.c. HR-pQCT: decrease in tibia vBMD and cortical thickness
Al Refaie et al. Italy [26]	T2DM Dulaglutide = 30 Semaglutide = 24	Observational study (52 weeks)	b-ALP CTX BMD-LS and BMD-FN and BMD-TH by DXA TBS BMD-LS and BMD-FN and BMD-TH by REMS	Body Weight: Dulaglutide: −4.3Kg ** Semaglutide: −3.4 Kg ** b-ALP: Dulaglutide: + 2.5 (µg/L) Semaglutide: + 2.3 (µg/L) CTX: Dulaglutide: +0.040 (ng/L) Semaglutide: +0.048 (ng/L) BMD-LS by DXA: −4.6% * TBS: +1.9% BMD-LS by REMS: −1.9% BMD-FN by DXA: −4.1%* BMD-FN by REMS: −3.8%* BMD-TH by DXA:−4.2%* BMD-TH by REMS: −3.7% *

*T2DM* type 2 diabetes mellitus, *Met* metformin, *b-ALP* serum bone alkaline phosphatase, *BMD* bone mineral density, *LS* lumbar spine, *FN* femoral neck, *TH* total hip, *DXA* dual energy X-ray absorptiometry. *CTX* C-terminal telopeptide of type 1 collagen, *TRAcP5b* tartrate-resistant acid phosphatase 5b, *GLP-1RAs* glucagon-like peptide-1 receptor agonists, *P1NP* N-terminal propeptide of type 1 procollagen, *WB* whole body, *HRpQCT* high-resolution peripheral quantitative computed tomography, *QCT* quantitative computed tomography, *T1DM* type 1 diabetes mellitus, *MOF* major osteoporotic fracture, *NTX* type 1 crosslinked N-telopeptide, *OP* osteoporosis, *DPP-4i* dipeptidyl peptidase-4 inhibitors, *TBS* trabecular bone score, *REMS* radiofrequency echographic multi spectrometry

Not significant change = n.s.c.

Statistically significant difference at *p < 0.05; **p < 0.01

### Bone Mineral Density (BMD)

A BMD by dual X-ray absorptiometry (DXA) scan was performed in ten studies, though one of these evaluated only whole body BMD [18]. High-resolution peripheral Quantitative Computed Tomography (HR-pQCT) was used in two studies [19, 25], while Radiofrequency Echographic Multi Spectrometry (REMS) was used in only one study [26]. In most studies, lumbar and femoral BMD by DXA showed very modest and non-significant increases [16, 19–21], or decreases [20, 23]. However, three recent studies have documented significant reductions in both BMD-lumbar spine and BMD-Total Hip in patients treated with GLP-1RAs (mainly semaglutide and dulaglutide); in all these studies, a significant reduction in body weight was also documented [24–26]. Moreover, Al Refaie’s study evaluated BMD using the REMS technology, which, in addition to assessing bone density, captures certain qualitative characteristics of the bone in T2DM patients [26, 27]. Furthermore, Hansen’s study documented that in subjects treated with semaglutide, volumetric bone mineral density (vBMD) and cortical thickness measured by HR-pQCT were reduced at the tibia but not at the radius [25]. In contrast, Hygum’s study found no significant changes in HR-pQCT parameters after 26 weeks of liraglutide therapy [19]. In the Hansen’s study, the microindentation values measured after 12 months of semaglutide therapy showed no significant differences compared to baseline [25].

### Bone Turnover Markers (BTMs)

**Fig. 2:**
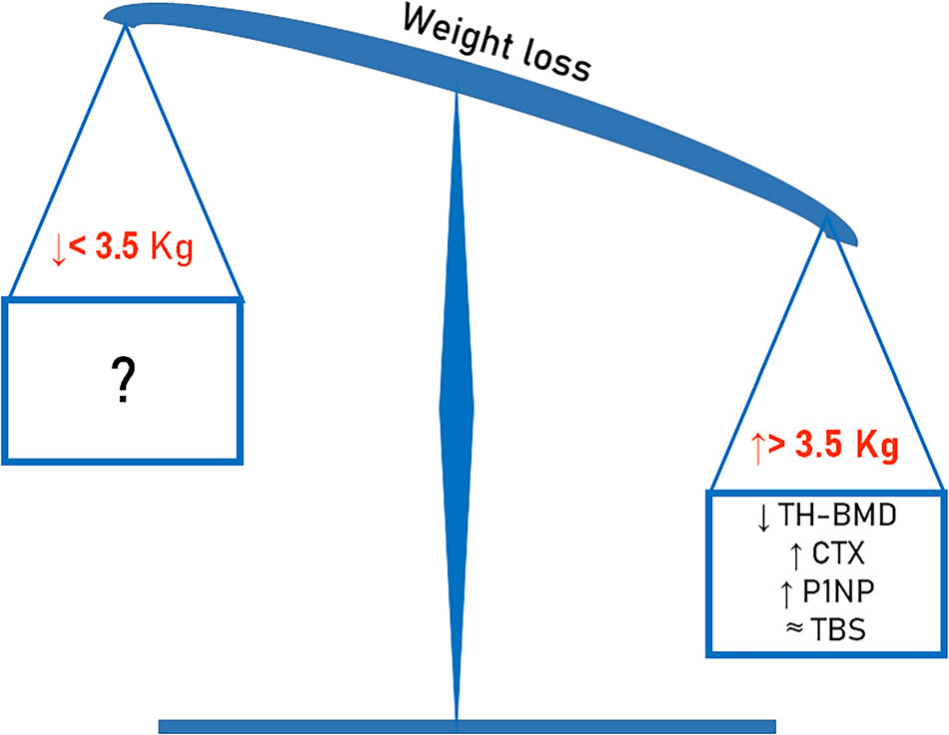
The weight loss in patients treated with Glucagon-Like Peptide-1 Receptor Agonists (GLP-1RAs) influences the change in the bone mineral density (BMD) and in the bone turnover markers (BTMs)

Changes in bone turnover markers were reported in only eight of the selected studies involving patients treated with GLP-1RAs. Specifically, bone resorption markers, such as N-terminal or C-terminal cross-linked telopeptide of type I collagen (NTX or CTX), were evaluated in six studies [15, 19, 21, 23, 25, 26]. A statistically significant increase in these markers was observed in studies that achieved a more pronounced reduction in body weight [25, 26] (Fig. 2). Bone formation markers, specifically bone alkaline phosphatase (b-ALP) and P1NP, were measured in seven studies. Five of these studies [15, 18, 19, 21, 23] reported no significant changes. More recently, Hansen’s study [25] observed a significant increase in P1NP, while Al-Refaie’s study [26] reported a significant increase in b-ALP. In both of these latter studies, patients exhibited a marked reduction in body weight (Fig. 2).

### Fractures

Only two studies address fracture risk [17, 22]. According to Driessen’s population-based cohort study, GLP-1RAs use, compared to other antihyperglycemic medications, is not associated with a reduced risk of bone fractures [17]. Al Mashadi’s study, which compares the risk of major osteoporotic fractures (MOFs) in patients on GLP-1RAs therapy versus those on dipeptidyl peptidase-4 inhibitor (DPP-4i) therapy, indicates that GLP-1RAs users have a significantly lower risk of hip fracture and a reduced risk of MOFs compared to DPP-4i users [22].

## Discussion

The class of GLP-1RAs drugs has garnered significant scientific and media attention, not only for its transformative impact on the treatment of diabetes mellitus and obesity, but also for its intriguing potential in cardiovascular, hepatic, and metabolic health. In contrast, the effects of GLP-1RAs on bone metabolism and bone mineral density remain less clearly defined. However, the studies analyzed in this review, despite their considerable heterogeneity, allow us to draw several important considerations. As previously introduced, in vitro and animal studies have shown that GLP-1RAs treatment promotes osteoblastogenesis and bone formation [6, 7] while inhibiting osteoclastogenesis and bone resorption [10, 11].

Clinical studies do not corroborate the preclinical data on bone formation and bone resorption. A significant increase in PINP was observed only in the Lepsen study, which included obese women who had previously followed a very low-calorie diet [28]. However, in Lepsen’s study the two bone formation markers, b-ALP and OC, did not show any increase [28]. Furthermore, a significant increase in bone formation markers (PINP and b-ALP) is evident only in the studies by Hansen and Al-Refaie, which documented a marked reduction in weight and an even greater increase in the bone resorption marker CTX [25, 26]. In clinical studies, the resorption markers CTX and NTX displayed a pattern opposite to that observed in preclinical studies. In fact, CTX showed a tendency to increase in all six studies that measured it, with a pronounced increase in studies where more substantial weight loss was observed [16, 25, 26]. The link between weight loss and increased bone resorption markers is supported by several studies in the literature [29]. Specifically, a study by Lepsen et al. demonstrated that even just 8 weeks of a low-calorie diet led to a significant increase in CTX levels in obese women [28]. Furthermore, in T2DM patients treated with GLP-1RAs, the increase in CTX may, at least in part, be attributed to the resumption of active osteoclastic activity, which had previously been suppressed by high glucose levels and the accumulation of advanced glycation end-products (AGEs) [1, 3]. Additionally, several interventional studies have reported that weight loss achieved through caloric restriction, with or without exercise, leads to increased levels of bone resorption markers and a reduction in BMD measured by DXA, with a more pronounced decrease at the total femur than at the lumbar spine [30]. The greater effect of weight loss on total femur BMD could reflect the overestimation of lumbar BMD resulting from artifacts due to aortic calcifications and degenerative changes or, more likely, be due to the greater sensitivity of femoral BMD to weight variations, linked to the different distribution of trabecular and cortical bone at the two skeletal sites [13]. Based on the analysis of the clinical studies reviewed, it appears that GLP-1RAs, particularly the most recent and potent ones, can significantly reduce body weight, increase bone turnover, and decrease BMD. These findings suggest a potential increase in fracture risk for individuals treated with GLP-1RAs. Additionally, it is well-established that weight loss achieved through intensive lifestyle interventions in overweight T2DM patients is associated with a reduction in BMD and a significant increase in fragility fractures [31, 32]. Therefore, the reduction of BMD in patients treated with GLP-1RAs who experienced significant weight loss further supports the “Mechanostat” theory, which suggests that bone mass and structure are influenced by mechanical load [14, 30, 33]. However, the data in the literature seem to exclude this risk; in fact, no study has reported an increase in fragility fractures in patients treated with GLP-1RAs [14, 34, 35]. Moreover, the meta-analysis by Cheng showed that GLP-1RAs treatment in patients with T2DM was associated with a lower risk of bone fracture and that this effect was more significant with a longer duration of treatment [36]. Zhang’s recent meta-analysis of 44 randomized controlled trials found that GLP-1RAs treatment may reduce the risk of fractures in T2DM patients, with the benefit becoming more pronounced with longer treatment durations, particularly beyond 18 months [37]. On the other hand, it is well-known that bone strength, and consequently the risk of fragility fractures, is not solely determined by BMD but also by other qualitative characteristics such as microarchitecture, trabecular bone, strength, and resistance. This is particularly true for patients with T2DM, who exhibit an increased risk of fractures despite having normal or even elevated BMD values [1, 3]. Some evidence from the literature suggests that GLP-1RAs may improve bone quality, particularly in patients with T2DM. It is well known that diabetic osteopathy is characterized by a significant reduction in bone turnover. Therefore, the pronounced increase in resorption markers and, to a lesser extent, in formation markers induced by GLP-1RAs could enhance bone structure and quality [1, 3]. Similarly, the assessment of BMD by using the REMS technology, which reflects qualitative characteristics of bone, does not show significant differences at the lumbar level following treatment with GLP-1RAs, unlike BMD measurement by DXA [27]. In this context, the trend of the trabecular bone score (TBS) observed in the study by Al Refaie is noteworthy [26]. The TBS, measured at the lumbar level, has been demonstrated to be a reliable index of bone microarchitecture and is considered more accurate than BMD in predicting fracture risk in patients with T2DM [38]. In Al Refaie’s study, conducted on T2DM patients, a 12-month treatment with dulaglutide or semaglutide resulted in a 4.6% reduction in lumbar spine BMD while producing a modest 1.2% increase in TBS. These findings support the hypothesis that GLP-1RAs may have a neutral or mildly positive effect on bone microarchitecture and quality [26]. The two studies that utilized QCT and HRpQCT to assess BMD and bone microstructure yielded quite discordant results [19, 25]. In fact, in the study by Hygum, a 26-week treatment with liraglutide did not result in significant changes in either lumbar and femoral volumetric BMD or radial and tibial HR-pQCT measurements [19]. Conversely, the recent randomized controlled study by Hansen, which compared 32 patients receiving semaglutide therapy for 12 months to 32 subjects receiving placebo therapy, reported a decrease in tibial vBMD and tibial cortical thickness in the semaglutide group compared with the placebo group, as assessed by HR-pQCT scans. However, the groups showed no differences in radial vBMD, radial cortical thickness, or estimated bone strength at the distal tibia or radius [25]. Additionally, the same study assessed bone material properties through impact microindentation using the OsteoProbe® (Active Life Technologies, Santa Barbara, CA, USA). This device measures the bone material strength index (BMSi) on the anterior surface of the tibial plateau, as previously outlined in international studies [39]. In the Hansen’s study, BMSi values measured after 12 months of semaglutide therapy showed no significant differences compared to baseline or the final values in the placebo group [25]. These data suggest that the reduction in BMD may represent an adaptation of the skeleton to lower mechanical loading following weight reduction, while the parameters related to bone quality and resistance do not exhibit any negative changes.

This narrative review has several limitations. First, clinical studies investigating the effects of GLP-1RAs therapies are limited in number and exhibit significant heterogeneity in terms of the types of GLP-1RAs studied, as well as variations in dosages and treatment durations. Second, the duration of the included studies does not exceed 52 weeks, with some lasting only 26 weeks, which hinders the ability to comprehensively assess changes in BMD and the incidence of fragility fractures. Moreover, MOFs are evaluated in only two studies. Third, most of the studies lack any information about participants’ physical activity levels, which is a key factor in bone health.

## Conclusions

Clinical studies have demonstrated that GLP-1RAs influence bone metabolism in a manner that contrasts with the findings of in vitro and animal studies, which predominantly reported an anabolic effect primarily mediated by the stimulation of osteoblastogenesis. Conversely, in humans, GLP-1RAs therapy has been shown to stimulate bone resorption, as evidenced by a significant increase in CTX levels, while promoting new bone formation to a lesser extent. In clinical studies, GLP-1RAs therapy, in diabetic patients, leads to a reduction in BMD, which is more pronounced at skeletal sites subjected to higher mechanical loading, such as the femur and tibia. This reduction appears to be associated with the degree of weight loss and may reflect a temporary adaptation of the skeleton to reduced mechanical loading following weight reduction. This hypothesis is supported by observations indicating that key parameters related to bone quality and strength (such as TBS, microindentation, HR-pQCT, and REMS) remain unaffected by GLP-1RAs therapy. Moreover, the incidence of fragility fractures does not increase and may even decrease. However, it is important to emphasize that the studies conducted so far are too short to draw definitive conclusions about the long-term effects of GLP-1RAs therapies on BMD and the risk of fragility fractures; therefore, further studies of longer duration (at least 2–3 years) are warranted.

## Data Availability

No datasets were generated or analysed during the current study.
